# Nanoselenium transformation and inhibition of cadmium accumulation by regulating the lignin biosynthetic pathway and plant hormone signal transduction in pepper plants

**DOI:** 10.1186/s12951-021-01061-6

**Published:** 2021-10-12

**Authors:** Dong Li, Chunran Zhou, Jinling Ma, Yangliu Wu, Lu Kang, Quanshun An, Jingbang Zhang, Kailin Deng, Jia-Qi Li, Canping Pan

**Affiliations:** 1 Innovation Center of Pesticide Research, Department of Applied Chemistry, College of Science, China Agricultural University, Beijing, 100193 China; 2 Institute of Food Science and Technology, Chinese Academy of Agricultural Sciences, Beijing, 100193 China

**Keywords:** Nanoselenium, Cd stress, Lignin biosynthetic pathway, Plant hormone signal transduction, Pepper plants

## Abstract

Selenium (Se) can promote the growth and resistance of agricultural crops as fertilizers, while the role of nano-selenium (nano-Se) against Cd remains unclear in pepper plants (*Capsicum annuum* L.). Biofortification with nano-Se observably restored Cd stress by decreasing the level of Cd in plant tissues and boosting the accumulation in biomass. The Se compounds transformed by nano-Se were primarily in the form of SeMet and MeSeCys in pepper tissues. Differential metabolites and the genes of plant signal transduction and lignin biosynthesis were measured by employing transcriptomics and determining target metabolites. The number of lignin-related genes (*PAL*, *CAD*, *4CL*, and *COMT*) and contents of metabolites (sinapyl alcohol, phenylalanine, *p*-coumaryl alcohol, caffeyl alcohol, and coniferaldehyde) were remarkably enhanced by treatment with Cd1Se0.2, thus, maintaining the integrity of cell walls in the roots. It also enhanced signal transduction by plant hormones and responsive resistance by inducing the biosynthesis of genes (*BZR1*, *LOX3*, and *NCDE1*) and metabolites (brassinolide, abscisic acid, and jasmonic acid) in the roots and leaves. In general, this study can enable a better understanding of the protective mechanism of nano-Se in improving the capacity of plants to resist environmental stress.

**Supplementary Information:**

The online version contains supplementary material available at 10.1186/s12951-021-01061-6.

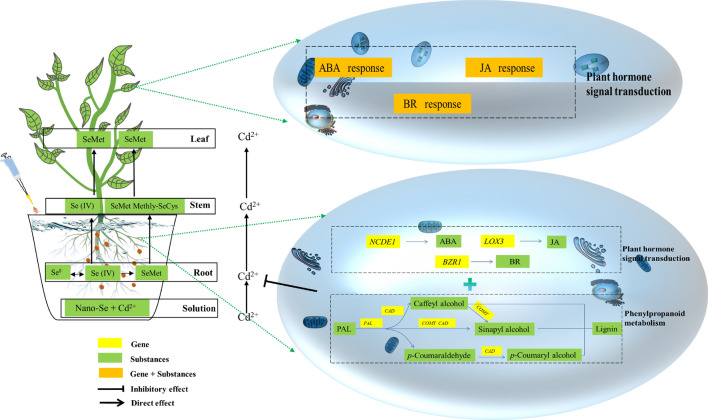

## Introduction

Heavy metal pollution in soil has become a global problem. The increase in absorption of Cd can lead to changes in plant metabolism, which limit crop productivity and pose a substantial threat to the environment. Concerns about the effects of Cd stress on crop physiology and biochemistry, the synthesis of plant primary and secondary metabolites, and even on the quality of agricultural products are widely expressed [1]. Existing research shows that the effects of Cd stress on crops are closely related to the organic matter in soil, cation exchange capacity, pH, soil microorganisms, secondary metabolites, crop species, and stage of growth [2]. The effects of Cd on different growth stages of crops have been studied using foliar sprays and polluted soil to evaluate the changes in crop physiology, biochemistry, enzymes, and non-enzymatic antioxidant systems. He et al. [3] have shown that Cd damages the metabolic function of plants by affecting photosynthesis and respiration, the absorption of nutrients and water, cell division, nitrogen metabolism, and the expression of proteins. Javed et al. [4] found that excessive Cd affects the activities of enzymes related to the Calvin cycle and carbohydrate metabolism, thereby inhibiting the growth of maize. Cd stress in plants stimulates the formation of reactive oxygen species (ROS), which cause protein oxidation, induce damage to lipids and nucleic acids, and change the metabolism of carbohydrates [5]. Therefore, Cd stress can affect the growth of crops by reducing photosynthesis and the synthesis of nutrients and may also regulate the synthesis of secondary metabolites through oxidative mechanisms.

Pepper fruits are rich in sugars, amino acids, vitamins, carotenoids, capsaicinoids, organic acids, flavonoids, phenolic acids, and volatile metabolites [6]. These metabolites determine the color, nutrition, and flavor of the pepper fruit, as well as its health functions, such as anti-oxidative, lipid-lowering, and anti-inflammatory properties. Capsaicinoids, which are unique to pepper, have useful clinical applications, including anti-cancer, analgesic, and health care effects [7]. The accumulation and transfer of metals in vegetables depend on environmental factors and plant genotypes. Vegetables easily take up heavy metals and accumulate them in their edible parts [8]. These compounds accumulate in different tissues and transform into a harmful composition of the food chain. Thus, it is highly important to research the effect of Cd stress on plant primary and secondary metabolites in peppers.

Plants are constantly faced with various types of infections in their growth, and a series of defense mechanisms have been formed during the long-term process of evolution. Exogenous stimulating factors, such as phytohormones, nanoengineering materials, and trace elements among others, have been widely studied in many crops for their ability to regulate plant growth and enhance the resistance of plants to stress [9, 10]. Javed et al. [4] found that exuded organic acids improve the absorption of nutrients and increase the content of antioxidant enzymes, such as superoxide dismutase (SOD) and peroxidase (POD), thereby increasing the tolerance of corn to Cd. Xu et al. [11] found that biochar can reduce the accumulation of Cd in pepper roots that had been subjected to a low level of Cd. The content of malondialdehyde decreased in the improved soil, and the content of chlorophyll increased significantly. Yan et al. [12] found that low concentrations of methyl jasmonate (0.1 µmol/L) can increase the contents of antioxidant enzymes (SOD, POD, and ascorbate peroxidase (APX) and jasmonic acid (JA), which reduce the harmful effects of Cd stress on pepper plants. Guo et al. [13] showed that treatment with Cd (100 mg/kg) and Si (400 mg/kg) positively correlated with the increased secretion of phenolic compounds in the rhizosphere. The exudation and accumulation of phenolic substances in plant tissues can alleviate Cd stress by chelating heavy metals or resisting oxidative damage. In addition, nanomaterials can penetrate plant cell walls to enter the plant and promote the healthy growth of plants by regulating the expression of related genes. Different nanomaterials have a substantial potential to reduce the toxicity of Cd but have no obvious negative effects on the growth of tomatoes, cucumbers, lettuce, and carrots [14].

Selenium, as a beneficial element for plant growth, promotes the growth of plants by increasing the accumulation of carbohydrates and plant hormones, which has attracted extensive research on the protection of plants from biotic and abiotic stresses. Se resulted in the production of low-mobility Cd compounds and increased the ability of cell walls to bind exogenous compounds in canola plants. It can reduce the level of Cd in the roots, affect the transportation of Cd to the stem, and further ameliorate the accumulation of Cd in the seed [15]. Liu et al. [16] suggested that organic amendments and Se promote the conversion of soil Cd from a bioavailable form to an immobilized one, thereby reducing the level of Cd in cereals. Se (5 µM) alleviated the Cr and methylglyoxal-induced oxidative damages and phytotoxicity by enhancing essential amino acids, antioxidants, and the glyoxalase system in *Brassica napus* L. [17]. It is effective at reducing the toxicity of heavy metals and limiting the absorption and translocation of heavy metals in plants. Se can also activate antioxidant enzymes and improve photosynthesis to quench the production of reactive oxygen species (ROS) under heavy metal conditions. However, the exact mechanism of secondary metabolism that results in the induction of tolerance to Cd stress by Se has not yet been elucidated. To date, various studies have elaborated the impact of Se on growth, primary metabolites, distribution and morphology, antioxidant systems, secondary compounds, and plant hormones [18, 19]. However, to our knowledge, there has not been a systematic study on the effect of nano-Se on the relationship of plant hormones and phenylpropanoid metabolism, particularly on the metabolites in the lignin synthesis pathway of pepper plants under Cd stress.

In our study, the growth, root ultrastructure, saccharide compounds, and Se speciation were determined, and we explored the changes in these indices using different concentrations of nano-Se in pepper plants under Cd stress. The key differential metabolites and genes of the lignin biosynthetic pathway and signal transduction by plant hormones were screened utilizing transcriptomics and measuring the levels of target metabolites. The related signal regulatory network was constructed to reveal the mechanism of intervention of nano-Se in the alleviation of Cd stress.

## Materials and methods

### Plant cultivation and experimental conditions

Hydroponic experiments were performed in a greenhouse at China Agricultural University, Beijing, China. It was conducted under the following conditions: 25/15 °C (day/night), photon flux with 225–300 µmol m^−2^ s^−1^, and a 14/12 h (light/dark) photoperiod with 75–80% humidity. Pepper seeds (*Capsicum annuum* L.) were germinated while watered with deionized water for 7 days. Uniform and healthy seedlings were transplanted into a plastic pot (2 L) and cultured in Hoagland-Arnon nutrient solution [20]. A half-strength nutrient solution was supplied for three days, and a full-strength solution was used until harvest. The solution was continuously refilled and renewed every two days to ensure an adequate supplementation of nutrition. The plants were pretreated in the nutrient solution for 10 days.

Se (0, 0.2, and 1 mg/L) and Cd (0 and 1 mg/L) were added to the vessel in form of Cd^2+^ in water/medium: 5% nitric acid (1000 mg/L) and nano-Se. The nano-Se was characterized as previously described [21]. The experiment was established with five repetitions of the six treatments that included Cd0Se0, Cd0Se0.2, Cd0Se1, Cd1Se0, Cd1Se0.2, and Cd1Se1. Samples were collected following continual treatment for 7 days. The roots were immersed in a 20 mmol/L solution of EDTA-Na_2_ for 5 min to remove the residual metal ions on the root surface. These plants were divided into roots, stems, and leaves after they were washed with deionized water. Some samples were used to determine the elements and metabolites. A portion of the samples was stored at − 80 °C for transcriptomics and the determination of genes.

### Analyses of saccharide compounds

The samples (20 mg) were extracted with 1 mL of ultrapure water. The mixed solution was subjected to ultrasound for 30 min and then centrifuged for 10 min at 14,000 rpm. All of the supernatant was filtered with a 0.1 μm injection syringe. The liquid was then diluted 20 times with ultrapure water. The concentration of saccharide compounds was determined using a Thermo Scientific Dionex ICS-5000^+^ ion chromatograph (Waltham, MA, USA). It was equipped with an SP single pump, EG eluent generator, AS-AP automatic sampler, DC electrochemical detector, and Chromeleon7.2 SR5 chromatographic data analytical software. The flow rate was 1 mL/min, and the injection volume was 10 µL. Ultrapure water and 200 mM NaOH (50% of NaOH was diluted with ultrapure water) were used as mobile phases A and B, respectively. The gradient elution procedure was as follows: 91% A, 0 min; 91% A, 18 min; 0% A, 21 min; 0% A, 31 min; 91% A, 32 min; and 91% A, 40 min.

### Transmission electron microscopy

The roots were merged in a 2.5% (v/v) solution of glutaraldehyde for 2 h at 4 °C. They were washed twice with phosphate-buffered saline (PBS, pH 7.2–7.4) at 4 °C for 15 min. The samples were added to 1% (v/v) OsO4 at 4 °C for 2 h and washed three times with PBS. The solution was dehydrated in a series of acetone solutions. Concentrations of 50%, 70%, 80%, and 90% of acetone were used to treat the samples for 15 min. The samples were treated twice with 100% of acetone for 10 min. The mixed solution was immersed overnight in the resin. It was polymerized at a high temperature and cut into slices of approximately 70 nm using a Lycra slicer (EM UC6; Leica, Wetzlar, Germany), dyed with uranyl acetate for 15 min, and treated with lead citrate for 10 min. The samples were dried and then photographed using a transmission electron microscope (TEM) (H-7650; JEOL. Ltd., Tokyo, Japan).

### Analysis of total selenium and cadmium

A mixed solution of HNO_3_ and HClO_4_ (9:1, v/v) was added to digest the plant root, stem, and leaf samples. They were diluted to 50 mL by adding deionized water into a volumetric flask. The supernatant was determined using atomic absorption spectrometry (HG-AFS; Haiguang, China) equipped with a hydride generation system to determine the concentration of Cd and Se in different tissues.

### Analysis of selenium speciation

One gram of the pepper tissues (roots, stems, and leaves) were hydrolyzed using 2 mL of Tris-HCl (75 mmol/L, pH 7.5)and 5 mg/mL of protease XIV (Sigma-Aldrich, St. Louis, MO, USA) at 37 °C. Finally, the supernatant was filtered through a 0.22–µm filter membrane. The samples were then subjected to high-performance liquid chromatography inductively coupled plasma mass spectrometry (HPLC-ICP-MS; SA-50, Thermo Scientific iCAP RQ).

The HPLC system was equipped with a Hypersil Gold C_8_ column (4.6 × 250 mm, 5 μm; Thermo, USA). A volume of 20 mM KH_2_PO4 that contained 0.5% heptafluorobutyric acid and methanol were used as mobile phases A and B, respectively. The flow rate was set at 1.0 mL/min. The gradient elution procedure was as follows: 100% A, 3 min; 95% A, 3.1 min; 95% A, 14 min; 100% A, 14.1 min; and 100% A, 15.0 min. The mass spectrometer conditions were as follows: the radio-frequency power was 1550 W; the plasma gas flow rate was 14 L/min; the flow rate of carrier gas was 1.06 L/min; the flow rate of auxiliary gas flow was 0.8 L/min; the dwell time was 0.1 s, and the analysis mass number was 78 Se. Five standard selenoamino acids were used in this experiment and included the following: Se(IV) (selenite), Se(IV) (selenite), SeCys (selenocysteine), MeSeCys (methylselenocysteine), and SeMet (selenomethionine) purchased from the National Institute of Metrology for Certified Reference Materials, Beijing, China.

### Plant hormone analyses

The method is a modification of previous research [22]. The roots and leaves were ground to a powder in liquid nitrogen. The samples (0.1 g) were then subjected to 1 mL of extraction solution that contained methanol: water: formic acid (80:19:1, v/v/v) using ultrasound for 10 min. They were centrifuged for 5 min at 12,000 rpm. These supernatants were added to 50 mg of primary secondary amine (CNW Technologies GmbH, Shanghai, China) in a 2 mL centrifuge tube. The mixed solution was dried with nitrogen, and the volume was kept constant to 100 µL with an aqueous solution of 80% methanol. All of the supernatant was filtered using a 0.22-µm organic membrane. An Agilent 6465 Triple Quadrupole UPLC−MS/MS (Ultivo; Agilent Technologies, Santa Clara, CA, USA) was equipped with an EclipsePlus C18 column (2.1 × 50 mm, 1.8 μm). The flow rate was 0.4 mL/min. Acetonitrile and 0.1% formic acid in water were used as mobile phases A and B, respectively. The gradient elution procedure was as follows: 80% A, 0 min; 5% A, 4 min; 80% A, 4.1 min, and 80% A, 5.2 min. It used positive and negative ion scanning mode and multiple reaction monitoring (MRM). All the parameters of MRM and collision energy (Additional file 1: Table S1) were optimized for maximum selectivity and sensitivity.

### Determination of the metabolites related to lignin synthesis

A total of 0.1 g of roots was ground to a powder in liquid nitrogen. The samples were extracted using 1 mL of an aqueous solution of 80% methanol (1% formic acid). These mixtures were shaken for 3 min, treated with an ultrasonic instrument for 10 min and centrifuged at 10,000 rpm for 5 min. The supernatant was added to a 2 mL centrifuge tube filled with 50 mg of C18 and 5 mg of multiwalled carbon nanotubes (MWCNTs) purchased from the Sinopharm Chemical Reagent Co. (Beijing, China). They were vortexed for 2 min and centrifuged for 5 min at 10,000 rpm at 4℃. All the supernatant was filtered using a 0.22-µm organic membrane.

An Agilent 6465 Triple Quadrupole UPLC−MS/MS (Ultivo, Agilent Technologies) was equipped with a Venusil Hilic (2.1 × 50, 1.8 μm). The flow rate was 0.25 mL/ min. Acetonitrile and 0.1% formic acid water were used as mobile phases A and B, respectively. The gradient elution procedure was as follows: 5% A, 0 min; 95% A, 4 min; 5% A, 4.1 min; and 5% A, 5.2 min. It used positive and negative ion scanning mode and MRM. All the parameters of MRM and collision energy (Additional file 1: Table S2) were optimized for maximum selectivity and sensitivity.

The HPLC−UV (Agilent Technologies) was equipped with a Venusil Hilic (4.6 × 100, 5 μm). The flow rate was 1 mL/min. Acetonitrile and ultrapure water were used as mobile phases A and B, respectively. The compounds were eluted from a 5 min isocratic run at a 60:40 ratio of A to B. The UV absorption was monitored at 217 nm.

### mRNA expression analysis

#### Transcriptome sequencing

The roots and leaves were stored at − 80 °C and then used with an RNAprep pure Plant Kit (Tiangen Biotech, Beijing, China) to extract the total RNA following the manufacturer’s instructions. The concentration of RNA in different tissues was measured using an RNA Nano 6000 Assay Kit (Thermo Fisher Scientific, Wilmington, DE, USA). The Agilent Bioanalyzer 2100 system to assess the integrity of RNA (Agilent Technologies). A NEBNext Ultra^TM^ RNA Library Prep Kit for Illumina (NEB, Beverly, MA, USA) was used to generate sequencing libraries following the manufacturer’s recommendations, and index codes were added to the attribute. EdgeR was used to perform an analysis of the differential expression of two samples. Significant differential expression was established as the threshold for the Fold Change ≥ 1.5 and P < 0.01. The GOseq R packages based on Wallenius non-central hyper-geometric distribution implemented Gene Ontology (GO) enrichment analysis of the differentially expressed genes (DEGs) that adjusted for gene length bias in the DEGs. A pathway enrichment analysis was performed using KOBAS software to test the statistical enrichment of DEGs in the Kyoto Encyclopedia of Genes and Genomes (KEGG) pathways.

### Real-time quantitative PCR

Ten representative genes were selected to verify the transcriptomics results using quantitative real-time reverse transcriptase–PCR (qRT-PCR). The RNA extraction utilized an RNAprep pure Plant Kit according to the manufacturer’s instructions. The cDNA synthesis used a FastQuant RT Kit. The qRT-PCR was performed with SuperReal PreMix Plus (SYBR Green). The expression of the β-actin served as a reference. The primers for sequences (Additional file 1: Table S3) were purchased from Sangon Biotech (Shanghai, China). The specific methods used were previously described [22].

### Statistical analysis

A difference analysis was performed by a one-way analysis of variance (ANOVA) using SPSS 26.0 (IBM, Inc., Armonk, NY, USA). The graphs were constructed in Origin 2021 (OriginLab, Northampton, MA, USA.) and GraphPad Prism Version 8.0 (San Diego, CA, USA). Tukey’s *t*-test was used to evaluate the separation of means, and significant differences were detected at a p < 0.05.

## Results

### Nano-Se improves the biomass and saccharides under Cd stress

**Fig. 1:**
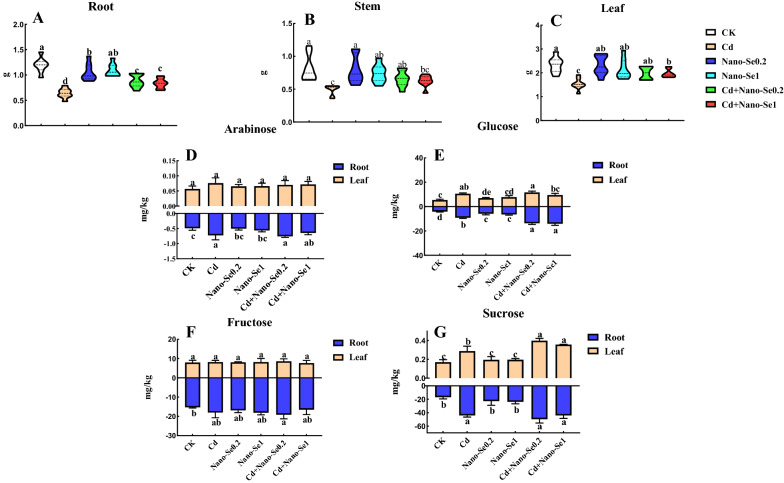
Effect of the interaction of nanoselenium (nano-Se) and cadmium (Cd) on the biomass and saccharides of pepper plants. The effects of different ratios of nano-Se (0, 0.2, and 1 mg/L) and Cd (0 and 1 mg/L) on the biomass (**A**–**C**), arabinose (**D**), glucose (**E**), fructose (**F**), and sucrose (**G**) in the leaves and roots of pepper plants. Different letters across treatments indicate significant differences at *P* < 0.05

Compared with the control, the addition of Cd significantly decreased the biomass of roots, stems, and leaves at the seedling stage (Fig. 1A–C). The co-application of Cd and nano-Se significantly improved the biomasses of pepper tissues compared with the Cd treatment. Cd1Se0.2 and Cd1Se1 enhanced the biomass of roots (38% and 30%), stems (33% and 26%), and leaves (30% and 29%) compared with the Cd treatment, respectively. However, the biomass of pepper tissues treated with the combination of Cd and nano-Se was less than those of the control and nano-Se alone.

The change of saccharides in the roots of different treatments with the exception of the control was more obvious than that in the leaves (Fig. 1D–G). The saccharide compounds arabinose, glucose, fructose, and sucrose in the roots notably increased, while the arabinose and fructose levels in the leaves had no obvious difference compared with the control. Cd, Cd1Se0.2, and Cd1Se1 increased the content of arabinose by 48%, 55%, and 32% in the roots, respectively, while there was no significant difference between them. The changing trend of glucose and sucrose was the most obvious since they maintained the upward trend in different tissues compared with the control. The levels of glucose increased by 1.2-, 2.3-, and 2.4-fold compared with the control in the roots following treatment with Cd, Cd1Se0.2, and Cd1Se1, respectively. Cd1Se0.2 and Cd1Se1 enhanced the content of glucose by 94% and 101% in the roots relative to the Cd, respectively. Combinations of Cd and nano-Se in different proportions increased the levels of glucose in leaves by 95%, 115%, and 77% compared with the control. Cd, Cd1Se0.2, and Cd1Se1 also enhanced the contents of sucrose in the roots by 1.6-, 1.9-, and 1.6-fold, respectively, and by 69%, 135%, and 110% in the leaves compared with the control, respectively.

### Nano-Se alleviates the ultrastructural damage induced by Cd

**Fig. 2:**
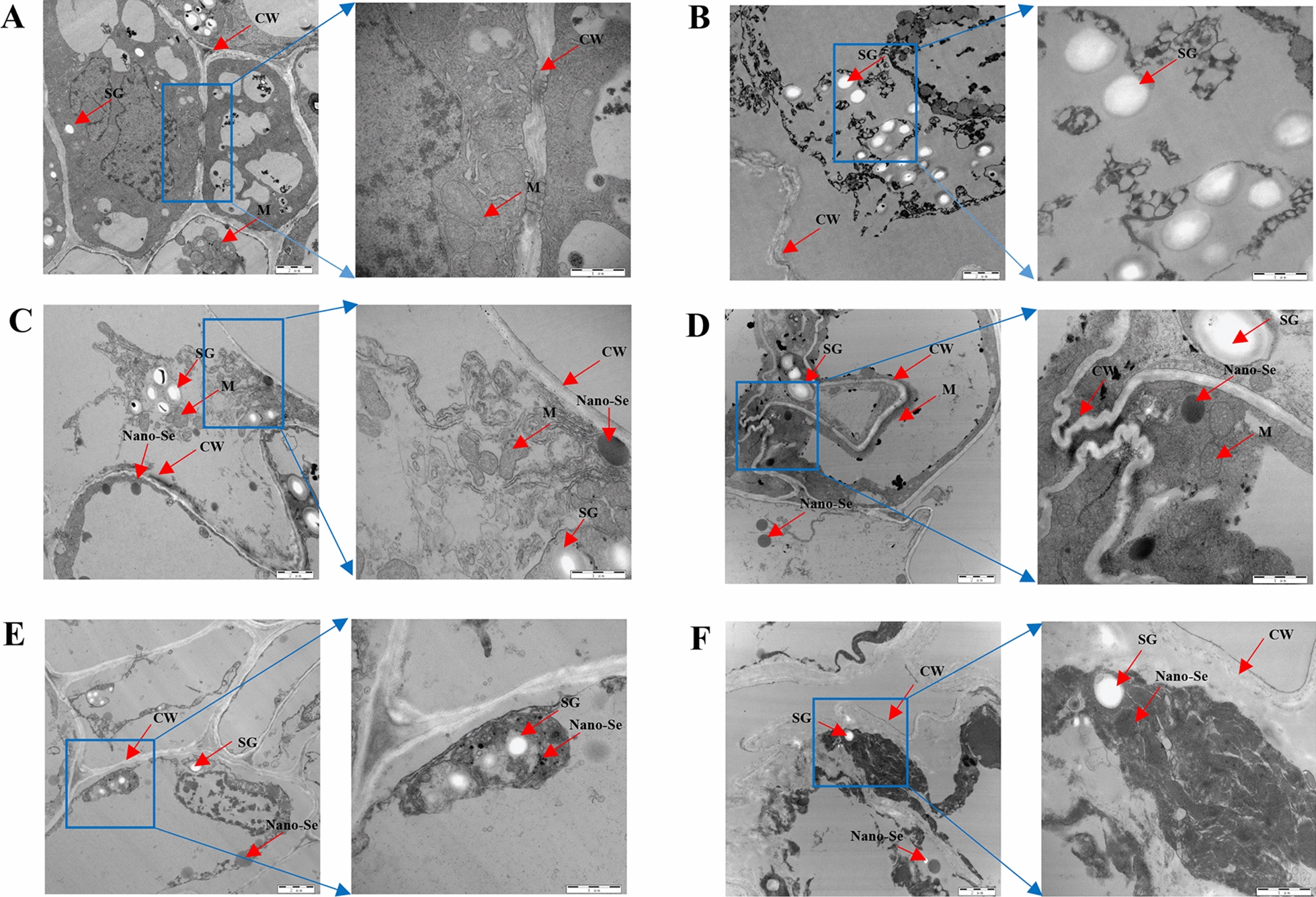
Effects of the control (**A**), Cd1 (**B**), Se0.2 (**C**), Se1 (**D**), Cd1Se0.2 (**E**), and Cd1Se1 (**F**) on the ultrastructure (CW, M, and SG) of roots by TEM. Cell wall: CW, M: mitochondrion, SG: starch granules. TEM: transmission electron microscopy

The ultrastructure within the root tip cells of pepper with different treatments is shown in Fig. 2. The root ultrastructure of control, nano-Se0.2, and nano-Se1 had clear mitochondria and cell walls (Fig. 2A, C, and D). The plants treated with nano-Se0.2 and nano-Se1 accumulated more selenium nanoparticles in the cell wall and inside the cell. However, Cd, Cd1Se0.2, and Cd1Se1 resulted in the enlargement of mitochondria and irregular cell walls in root tip cells compared with the control (Fig. 2B, E, and F). Compared with Cd alone, Cd1Se0.2 could restore the morphological damage of mitochondria and cell walls. In addition, the tissues treated with Cd1Se0.2 and Cd1Se1 accumulated fewer starch granules. As the concentration of nano-Se increased, the root tip cells were severely damaged, as manifested by incomplete cell walls and the rupture of mitochondria.

### Nano-Se affects the accumulation of Cd and total Se and the speciation of Se

**Fig. 3:**
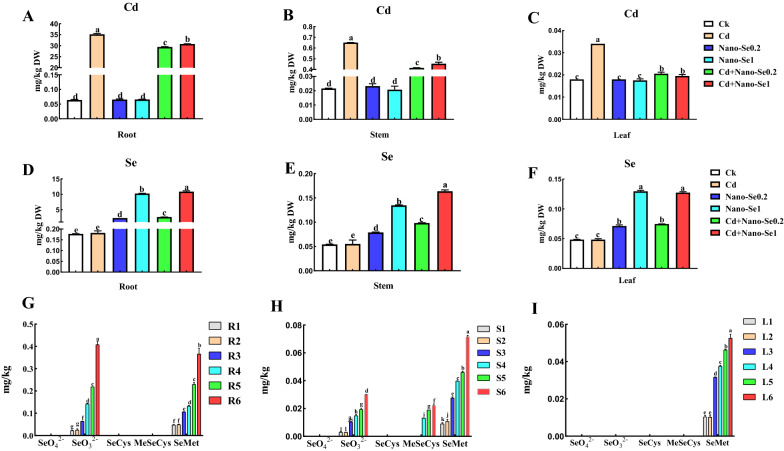
Effects of the different ratios of nano-Se (0, 0.2, and 1 mg/L) and Cd (0 and 1 mg/L) on the Cd (**A**–**C**), Se (**D–F**), Se speciation (**G–I**) in the leaves and roots of pepper plants. Different letters across treatments indicate significant differences at *P* < 0.05. Nano-Se: nanoselenium

Cd accumulated in the roots, stems, and leaves since the nutrient mixtures contained some Cd^2+^. As shown in Fig. 3A–C, the concentration of Cd was the highest in roots and was followed by the stems and leaves. The content of Cd in stems and leaves was much lower than that in the roots. The combination of nano-Se and Cd (Cd1Se0.2 and Cd1Se1) significantly decreased the levels of Cd in the roots, stems, and leaves of pepper plants than the Cd alone. The levels of Cd in the roots decreased by 17% and 13%, while those in the stems decreased by 36% and 30%. The levels in the leaves decreased by 40% and 42% in the Cd1Se0.2 and Cd1Se1 treatments, respectively, compared with the Cd treatment.

Different effects of treatments on the concentrations of Se in pepper tissues were examined (Fig. 3D–F). There was no significant difference in the Se content in pepper tissues without Se treatment. Increasing the concentration of nano-Se significantly increased the level of Se. Enzymatic hydrolysis was used to extract the Se species from the pepper tissues, and they were detected using HPLC–ICP-MS (Fig. 3G–I). The Se (IV), Se (VI), SeCys, MeSeCys, and SeMet were identified and quantified in different tissues. As a whole, the order of contents of Se speciation in the roots and aboveground parts of pepper was SeMet > Se (IV) > MeSeCys. SeMet was determined to be the predominant species in all of the pepper tissues. Se (IV) was also identified as the main form of Se in the roots and stems. A small amount of MeSeCys was also measured in the stems that had been treated with Se1, Cd1Se0.2, and Cd1Se1. Compared with the nano-Se0.2 and nano-Se1 treatments, the content of Se (IV) in the roots increased by 2.4- and 1.9-fold, respectively, and increased by 85% and 102% in the stems treated with Cd1Se0.2 and Cd1Se1, respectively. In addition, the levels of SeMet in the roots increased by 117% and 177%, by 67% and 80% in the stems and was enhanced by 46% and 40% in the leaves owing to the combination of Cd and nano-Se relative to the nano-Se0.2 and nano-Se1 alone, respectively.

### Nano-Se regulates plant hormones and the contents of related genes under Cd stress

**Fig. 4:**
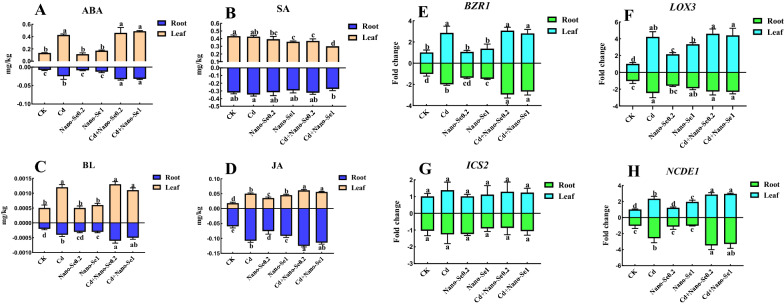
Effects of different ratios of nano-Se (0, 0.2, and1 mg/L) and Cd (0 and 1 mg/L) on the contents of plant metabolites related to hormones: ABA (**A**), SA (**B**), BL (**C**), JA (**D**), and the level of expression of the genes *BZR1*(**E**), *LOX3* (**F**), *ICS2* (**G**), and *NCDE1* (**H**) in leaves and roots of pepper plants. Different letters across treatments indicate significant differences at *P* < 0.05

As shown in Fig. 4A–H, these metabolites and genes were significantly altered in the root and leaves. Compared with the control and nano-Se alone, the contents of JA, ABA, and BL were remarkably enhanced, while the level of salicylic acid (SA) was apparently not affected by Cd, Cd1Se0.2, and Cd1Se1 in the roots/leaves. Cd1Se0.2 and Cd1Se1 increased the level of ABA (32/29%) in the root but had no apparent alteration in the leaves compared with the Cd treatment. Intervention with nano-Se0.2 enhanced the content of JA by 16% in the root and 22% in the leaves compared with Cd alone. Cd1Se0.2 and Cd1Se1 enhanced the content of BL by 103/48% in the roots but had no apparent changes in the leaves compared with the Cd.

The levels of expression of *BZR1*, *LOX3*, *ICS2*, and *NCDE1* of these genes related to metabolites were determined (Fig. 4E–H). The combination of different proportions of Cd and nano-Se caused significant changes in their expression in the roots and leaves, including *BZR1*, *LOX3*, and *NCDE1*. The expression of *ICS2* did not significantly change from the various treatments in the root and leaves. Compared with the Cd treatment, Cd1Se0.2 enhanced the content of *BZR1* in the root by 48% the level of *NCDE1* l increased by 35% in the root and 22% in the leaves.

### Nano-Se enhanced the accumulation of lignin-related metabolites and the levels of genes under Cd stress

**Fig. 5:**
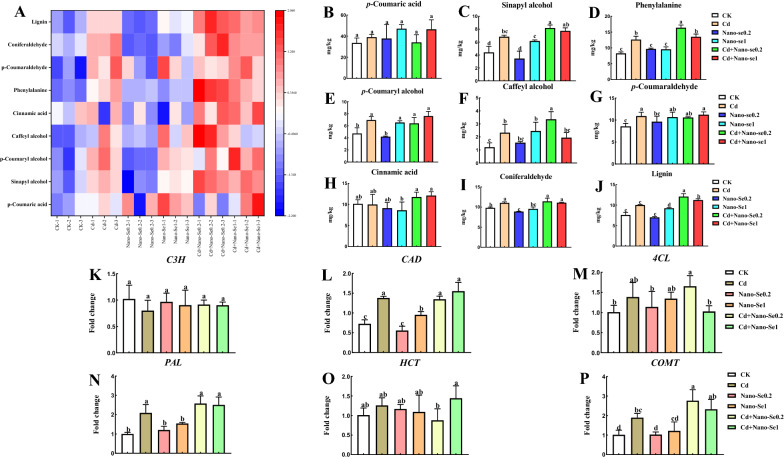
Effects of different ratios of nano-Se (0, 0.2, and1 mg/L) and Cd (0 and 1 mg/L) on the lignin biosynthesis-related metabolites and genes. A heatmap (**A**) was utilized to analyze the alteration of lignin-related metabolites. Levels of *p*-coumaric acid (**B**), sinapyl alcohol (**C**), phenylalanine (**D**), *p*-coumaroyl alcohol (**E**), caffeoyl alcohol (**F**), *p*-coumaraldehyde (**G**), cinnamic acid (**H**), coniferaldehyde (**I**), lignin (**J**); and the expression of *C3H* (**K**), *CAD* (**L**), *4CL* (**M**), *PAL* (**N**), *HCT* (**O**), and *COMT* (**P**) were determined in the roots of pepper plants. Different letters across treatments indicate significant differences at *P* < 0.05

As shown in Fig. 5A, compared with the control, the combination of different ratios of Cd and nano-Se regulated the lignin-related metabolites of phenylpropanoid metabolism, including *p*-coumaric acid, sinapyl alcohol, phenylalanine, *p*-coumaryl alcohol, caffeyl alcohol, *p*-coumaraldehyde, cinnamic acid, coniferaldehyde, and lignin. Compared with the Cd treatments, Cd1Se0.2 enhanced the levels of sinapyl alcohol (20%), phenylalanine (30%), and caffeyl alcohol (44%). The amount of lignin increased by 21% and 12% under Cd1Se0.2 and Cd1Se1 treatments in the root, respectively. However, the levels of *p*-coumaric acid and cinnamic acid had no apparent changes following their treatment.

The levels of expression of the lignin-related genes *C3H*, *CAD*, *4CL*, *PAL, HCT*, and *COMT* were measured (Fig. 5K–P). The treatments with Cd, Cd1Se0.2, and Cd1Se1 enhanced their levels of expression in the roots, including *CAD*, *PAL*, and *COMT*. *C3H*, *4CL*, and *HCT*, had no distinct alterations following treatment with Cd alone or a combination of Cd and nano-Se in the root. Compared with the Cd treatment, Cd1Se0.2 enhanced the expression of *COMT* by 46% in the roots.

### Nano-Se resulted in significant changes in the transcriptome of pepper root and leaves

**Fig. 6:**
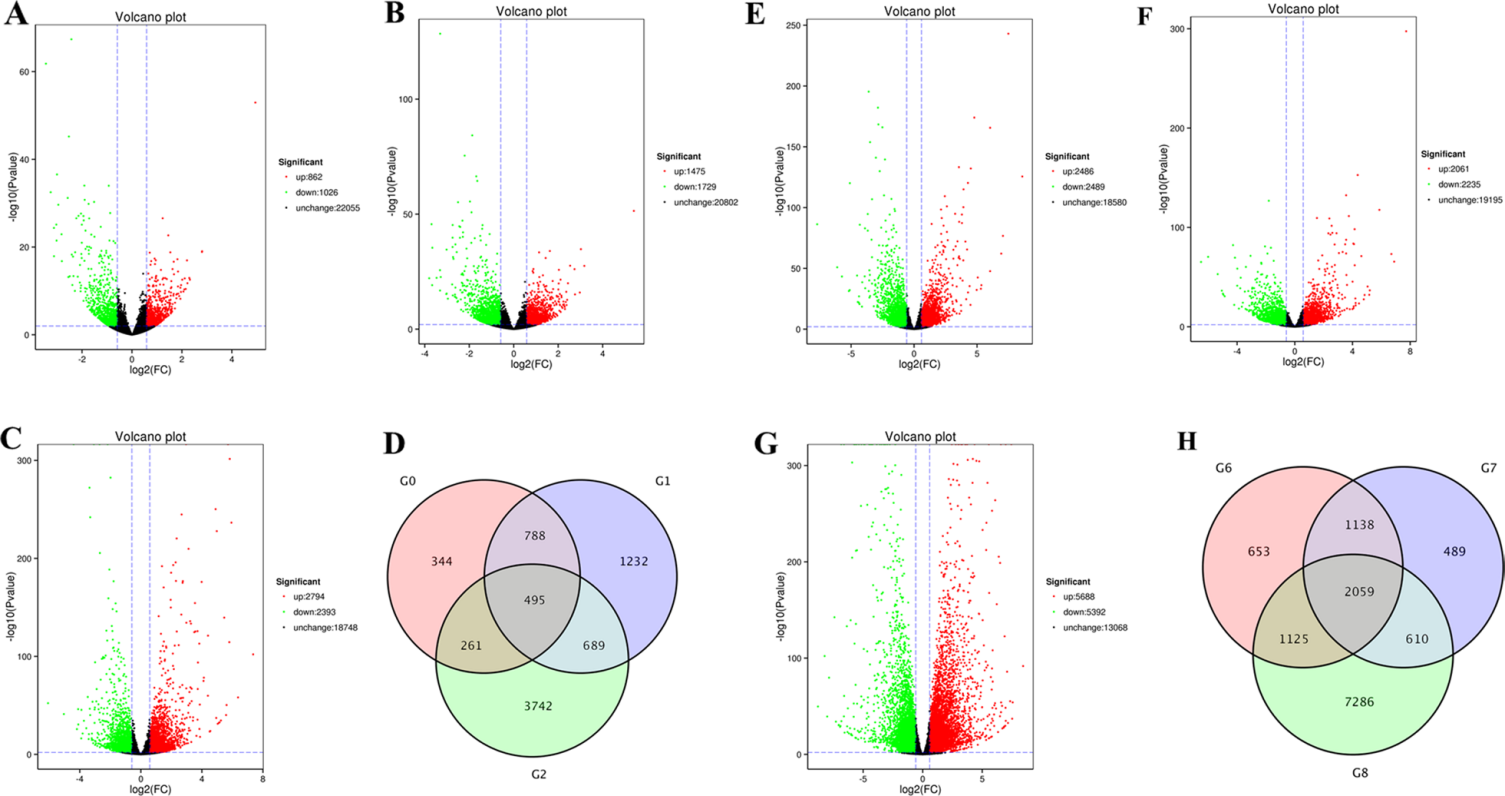
Volcano plots of differentially expressed genes (DEGs) for pairwise comparison: **A**, **E** CK and Cd1; **B**, **F** CK and Cd1Se0.2; **C**, **G** CK and nan-Se0.2 in the leaves and root. **D**, **H** DEGs overlapped in different treatments: CK and Cd (G0/G6); CK and Cd1Se0.2 (G1/G7); CK and nano-Se0.2 (G2/G8) in the leaves and roots

Four groups of pepper plants with different tissues (root and leaves) were analyzed to identify the transcriptional responses by treatment with nano-Se under Cd stress (Fig. 6). In the control and Cd treatment (G0 and G6), a total of 1888 DEGs were identified in the G0 groups; 862 were upregulated, and 1026 were downregulated relative to the control in leaves. A total of 4975 DEGs were identified in the G6 groups. A total of 2486 were upregulated, and 2489 were downregulated relative to the control in roots. In the control and Cd1Se0.2 treatments (G1 and G7), a total of 3204 DEGs were identified in the G1 groups, with 1475 upregulated and 1729 downregulated in the leaves. A total of 4,296 DEGs was identified in the G7 groups with 2061 upregulated and 2235 downregulated in the roots. A total of 5187 DEGs (up 2794 and down 2393) were identified in the control and nano-Se0.2 treatment (G2) in the leaves. A total of 11,080 DEGs (up 5688 and; down 5392) were identified in the control and nano-Se0.2 treatment (G8) in the roots. The DEGs of different treatments are shown in Additional file 1: Table S4.

**Fig. 7:**
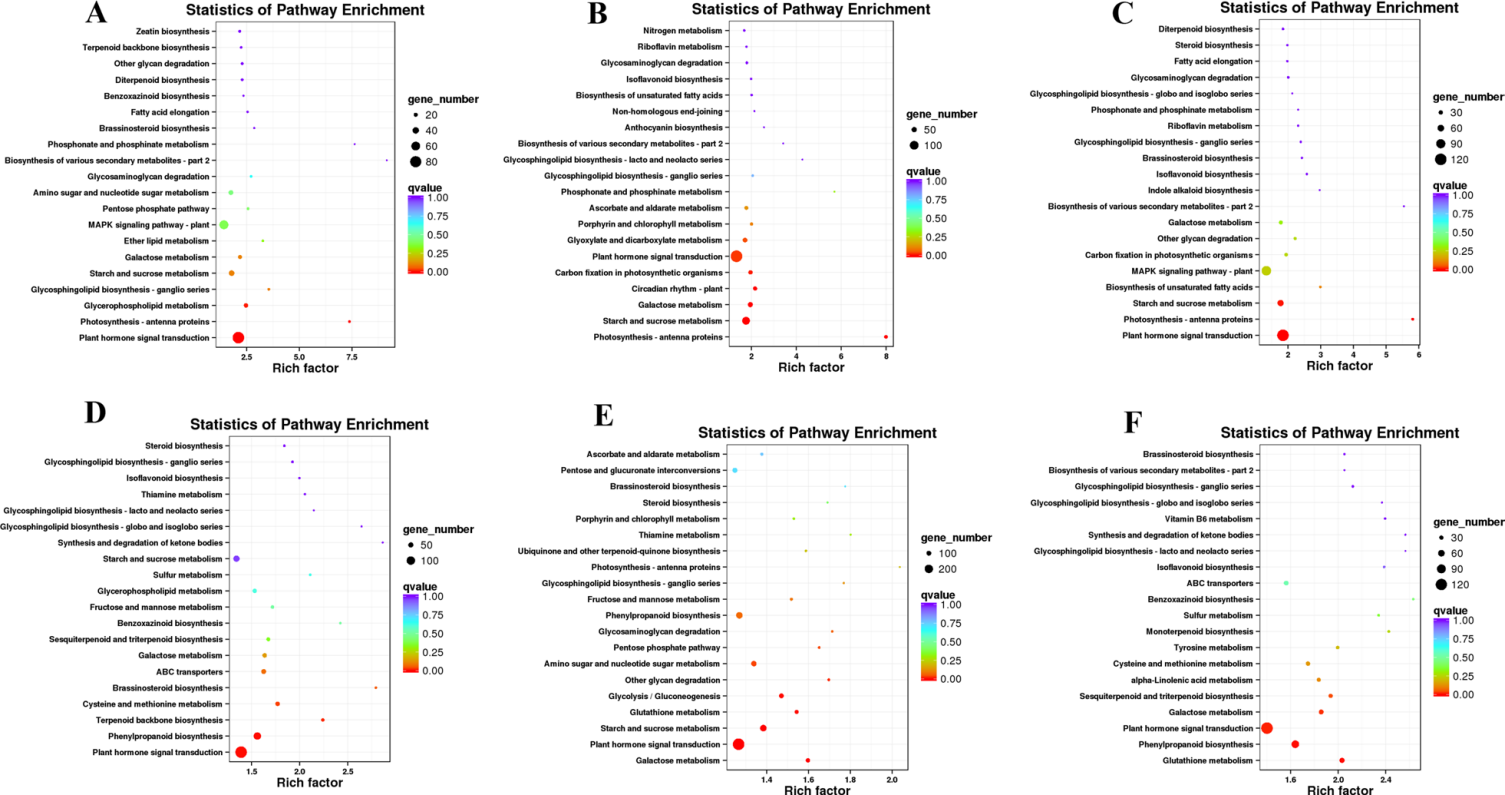
The top 20 enriched KEGG pathways of upregulated and downregulated in the different groups. The Q-value was represented with various colors as shown in the legend. The multiple hypothesis testing (range from 0 to 1) was corrected by Q-values and the *p*-value. KEGG, Kyoto Encyclopedia of Genes and Genomes

The GO and KEGG enrichment analysis was used to better understand the transcripts of pepper plants under Cd stress (Fig. 7). The DEGs were primarily classified into molecular functions, biological processes, and cell composition (Additional file 1: Figure S1 and S2). These DEGs in the leaves and roots of different treatments (CK vs. Cd, CK vs. Se0.2, and CK vs. Cd1Se0.2) indicated a significant enrichment in plant hormone signal transduction (Fig. 7A–C) by a KEGG-enriched analysis, while the phenylpropanoid biosynthesis was only markedly enriched in the roots (Fig. 7D–F).

## Discussion

**Fig. 8:**
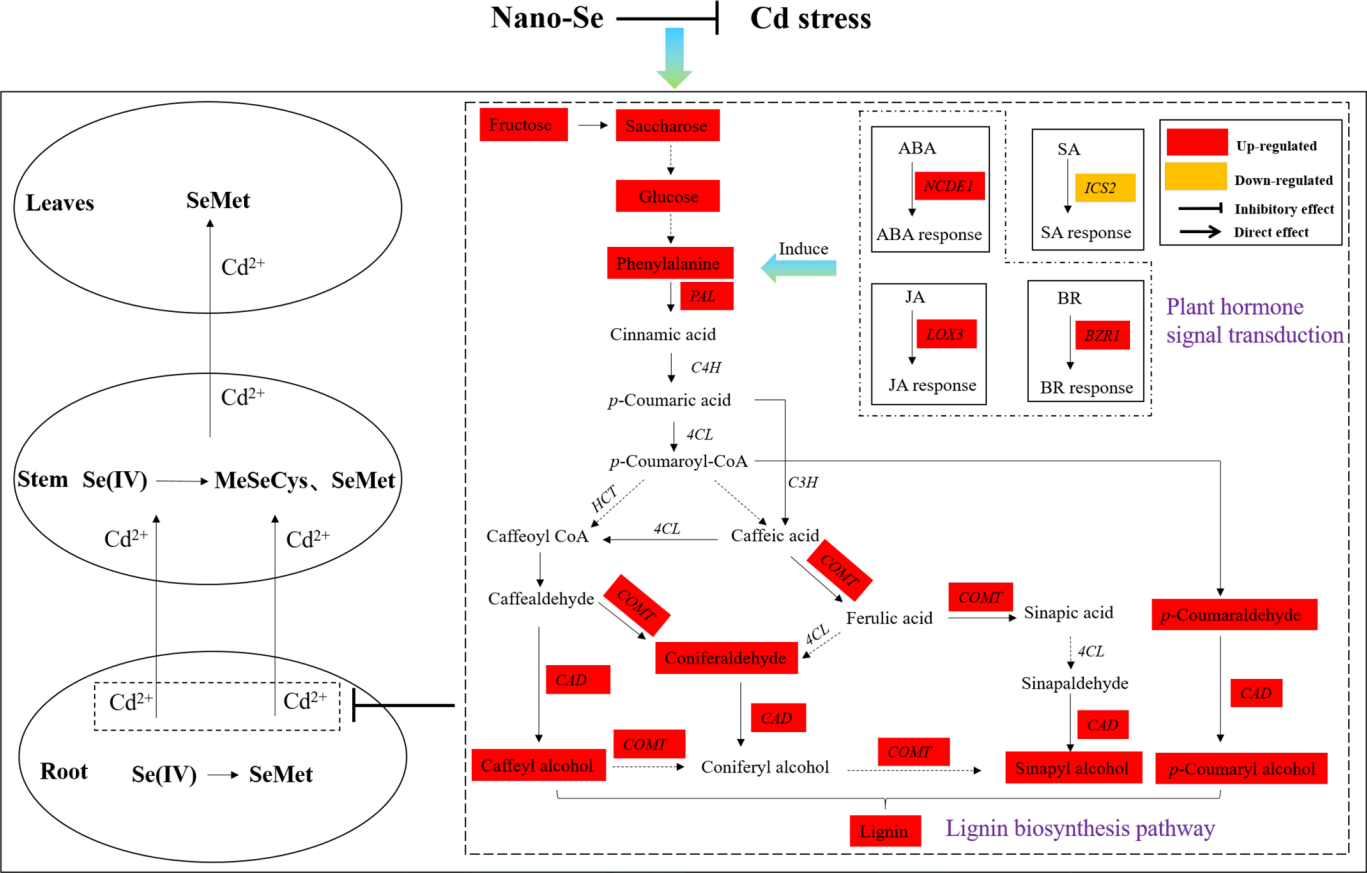
The proposed protective mechanism of nano-Se enhancing the resistance of pepper plants under Cd stress

Cd stress can hinder the growth of plants and cause a disorder of plant metabolism, thus, affecting the yield of crops. The minimization of Cd absorption in pepper plants is crucial to reduce the exposure to Cd toxicity and ensure food safety. The application of Se ameliorates the adverse effects of abiotic and biotic stress by adjusting different mechanisms, including the antioxidant system and secondary metabolism. However, few studies have been conducted on the effects of application of nano-Se on the mechanisms of resistance of pepper plants to heavy metal stress. In this study, the specific issues of nano-Se-induced plant growth and the associated absorption and transformation under Cd stress were examined. Nano-Se also stimulated the integrity of cell wall structure by regulating the lignin biosynthetic pathway in the root. It also improved the resistance by adjusting the signal transduction of plant hormones. These observations demonstrate that nano-Se enhances the tolerance of pepper plants to Cd stress that is compactly associated with the synergetic regulation of Se/Cd uptake and transformation, cellular homeostasis, and plant hormone/lignin-related signal pathway in pepper plants (Fig. 8).

The mass of roots, stems, and leaves of pepper plants decreased significantly under Cd stress (Fig. 1A–C). The accumulation of biomass decreased when the plants were subjected to an excessive accumulation of Cd. This could possibly be owing to interference with the uptake of carbohydrate compounds. In this study, it was observed that Cd in the pepper roots and leaves increased the levels of arabinose, sucrose, glucose, and fructose compared with the control and nano-Se treatments. However, a combination of Cd and Se (Cd1Se0.2 and Cd1Se1) can remarkably enhance the contents of glucose and sucrose compared with the treatment of Cd alone in the roots and leaves. Shahid et al. found that sodium selenate decreased the Cd and As levels in the plant, which helped to accumulate sugars and alleviate oxidative stress, thus, enhancing the nitrogen and carbon metabolism triggered by As and/or Cd stress [23]. The study of transcriptomics indicated that the starch and sucrose metabolism changed significantly in the leaves subjected to different treatments (G0, G1, and G2) but not in the roots (G6, G7, and G8). The accumulation of soluble sugar in leaves can help the plants to maintain water status and eliminate ROS under abiotic stress [24].

Selenium has been used as a base fertilizer or as a foliar spray to improve the content of Se in the edible parts of crops. The absorption of Se and its translocation and distribution in plants depends on the species of plant, mode of usage and concentration, physiological conditions, membrane transporter activity, and translocation mechanism [25]. In this study, the content of Se increased significantly with the increase in concentration of nano-Se. Most of the Se compounds transformed by nano-Se biofortification are selenoamino acids, primarily in the form of SeMet and MeSeCys. Several studies reported that the application of Se can reduce the absorption and transformation of plants and mitigate metabolic disturbances induced by heavy metals. Nano-Se inhibited the accumulation of Cd in the roots, and the amounts of Cd were significantly reduced in the upper parts of pepper. In addition, the content of Cd in the roots was greater than that in the stems and leaves (Fig. 2A–C). The possible mechanism could be to reduce the accumulation of Cd in plants tissues by binding Cd and converting it into other non-absorbable forms. This experiment also indicated that nano-Se absorbed by the roots can be quickly transformed into an organic state (SeMet) in the roots. Selenocompounds could form a complex with Cd, which could effectively retain Cd and inhibit its upward translocation. This phenomenon is also reflected in other crops, including rice (*Oryza sativa* L) [26, 27] and bok choy (*Brassica chinensis*) [28].

Few studies have examined the influence of nano-Se on the metabolites and genes related to lignin synthesis in the phenylpropanoid pathway of pepper plants. Previous research showed that the *PAL*, *C3H*, *CAD*, *4CL*, *HCT*, and *COMT* genes encode compounds in the phenylpropanoid pathway that can lead to lignin biosynthesis in plants. The levels of these genes positively correlate with the lignin levels of pepper plants. Lignin biosynthesis supports plant growth and the transportation of water and nutrients. As shown in Fig. 5, the contents of *PAL*, *CAD*, *4CL*, and *COMT* increased significantly following treatment with the combination of different proportions of Cd and nano-Se. Cd1Se0.2 was the most effective. Studies have found that the level of *PAL* was upregulated during lignin biosynthesis in tobacco [29]. The lignin biosynthetic gene of *4CL* is activated by MYB transcription factors, which induce lignin synthesis. The levels of *C3H*, *CAD*, *HCT*, *4CL*, and *COMT* were upregulated in lignin biosynthesis, which resulted in the accumulation of lignin and thickened cell walls, thus, enhancing osmotic protective agents and mitigating the effects of salt in apple [30]. The formation of *p*-coumaryl alcohol, caffeyl alcohol, and coniferyl alcohol is catalyzed by cinnamyl alcohol dehydrogenase in the biosynthesis of monolignol [31]. Since plants are continually developing in response to a changing environment, fixation by plants reprograms the distribution of metabolites to maintain vigorous growth and survival [32]. Nano-Se biofortification also significantly alleviated the damage induced by Cd owing to improvements in the ultrastructural integrity of the plasma membrane and mitochondria in the root tip, which improved the growth of pepper plants (Fig. 2). Zhao et al. suggested that Se improved the morphology of roots and regulated the absorption of nutrients by the roots. This could play a comprehensive role in reducing the absorption of Cr by roots, thus, reducing Cr stress and maintaining plant growth [33]. In our study, different ratios of Cd and nano-Se (Cd1Se0.2 and Cd1Se1) were more effective than Cd/Se alone in enhancing the levels of sinapyl alcohol, phenylalanine, *p*-coumaroyl alcohol, caffeoyl alcohol, and coniferaldehyde in the pepper root, which result in the induction of lignin biosynthesis (Fig. 5A–J). These metabolites that were upregulated in the lignin biosynthetic pathway are regarded as a determinant of lignin characteristics, which are conducive to the engineering of lignin to improve the development and utilization of plants [34]. Cui et al. indicated that Se reduced the level of ROS and enhanced the lignin content and thickness of the cell walls under Cd stress, resulting in an enhancement of the mechanical force of the cell walls by inducing the expression of lignin synthesis and Cd-related genes [35]. Therefore, we conclude that the application of nano-Se induced the biosynthesis of lignin-related genes and metabolites, thereby enhancing the mechanical strength of the cell wall and inhibiting the cellular absorption of Cd.

Importantly, the addition of nano-Se can regulate signal transduction by plant hormones and coordinately affect the lignin metabolic pathway under Cd stress. Plant hormones play an important role in coordinating responses to environmental change and are essential for stress tolerance and the yield of crops [9]. The root morphology and metal transporters were changed by adjusting the plant hormones and allocating the nutrient components [36]. Foliar spray plant hormones, such as JA, SA, and BR, improve the resistance, attributes of quality, nutrients, and shelf life in different crops [37, 38]. In our study, Cd1Se0.2 can significantly increase the expression of genes related to plant hormones in the roots and leaves compared with those of other treatments, including *BZR1*, *LOX3*, and *NCDE1*. These genes also upregulated the plant hormones BR, ABA, and JA in the root and leaves under Cd stress. In our previous study, nano-Se significantly increased the contents of genes related to JA and ABA in pepper fruit [22]. Low MeJA concentrations can mitigate the adverse effect of Cd by significantly increasing the level of JA observed at 48 h under 50 mg/L Cd and 50 mg/L Cd+0.1 mmol/L MeJA treatment in the *Capsicum frutescens* var. *fasciculatum* seedlings [12]. JAs enhance the defense of the crop against water loss by producing more osmotic compounds, such as cuticles, proline, lignin, waxes, and polyphenols [39]. JAs play an important role in the induction of gene expression and the increase in PAL activity, which is the primary enzyme in the pathway that produces phenolic compounds [39]. The mechanism of BRs mitigates heavy metal toxicity by boosting the antioxidant system and expression of MAPK and increasing the levels of proline, antioxidants, potassium, sodium ions, and osmolytes [40, 41]. ABA mitigated the adverse effects of Cu stress and protected the glandular trichomes, which improved the development and oxidative stress tolerance of plants by adjusting antioxidant defenses [42]. They synergistically regulate plant metabolism and enhance stress resistance through the interaction of SA, JA, and ABA [43]. In addition, diverse phytohormone signal pathways, such as JA, can also regulate phenylpropanoid metabolism [32].

## Conclusions

The diagram illustrates the potential of nano-Se in reducing the toxicity of Cd in pepper plants (Fig. 8). In this study, treatment with Cd alone observably inhibited growth, the accumulation of biomass, and absorption of nutrients. It also severely impaired the cellular wall integrity and the structure of mitochondria. Nano-Se biofortification markedly ameliorates the damage induced by Cd by reducing its content and increasing the accumulation of biomass in the form of glucose and sucrose in the roots and leaves. The Se compounds transformed by nano-Se were primarily in the form of SeMet and MeSeCys. The key differential metabolites of plant signal transduction and phenylpropanoid metabolism were screened by utilizing transcriptomics and determining the target metabolites. The levels of expression of lignin-related genes (*PAL*, *CAD*, *4CL*, and *COMT*) and the contents of metabolites (sinapyl alcohol, phenylalanine, *p*-coumaryl alcohol, caffeyl alcohol, and coniferaldehyde) in the roots were observably increased by treatment with Cd1Se0.2, resulting in an enhancement of thickness of the cell walls. It also improved the stress-responsive resistance by inducing the genes involved in plant signal transduction (*BZR1*, *LOX3*, and *NCDE1*) and the metabolites (BR, ABA, and JA) in the root and leaves. Overall, this study provides an enhanced understanding of the protective effect of Se in managing abiotic stress and improving the ability of plants to resist environmental stress.

## Supplementary Information

**Additional file 1: Table S1.** UPLC-MS/MS parameters of plant hormones; **Figure S1.** GO class of different treatments in the root; **Table S2.** UPLC-MS/MS parameters of lignin-related metabolites; **Figure S2.** GO class of different treatments in the leaves; **Table S3.** Primer sequences for qPCR; **Table S4.** Statistics of the number of differentially expressed genes.

## Data Availability

All data generated or analyzed during this study are included in this published article within each editable graph.
