# Supplementary material for: Nanoselenium transformation and inhibition of cadmium accumulation by regulating the lignin biosynthetic pathway and plant hormone signal transduction in pepper plants
Source: J Nanobiotechnology. 2021 Oct 12;19:316. doi: 10.1186/s12951-021-01061-6 (PMC8507250; doi:10.1186/s12951-021-01061-6)
Supplement: Supplementary file 1 — Additional file 1: Table S1. UPLC-MS/MS parameters of plant hormones; Figure S1. GO class of different treatments in the root; Table S2. UPLC-MS/MS parameters of lignin-related metabolites; Figure S2. GO class of different treatments in the leaves; Table S3. Primer sequences for qPCR; Table S4. Statistics of the number of differentially expressed genes. [file 12951_2021_1061_MOESM1_ESM.docx]

Nanoselenium transformation and inhibition of cadmium accumulation by regulating lignin biosynthesis pathway and plant hormone signal transduction in pepper plants

Dong Lia, Chunran Zhoua, Jinling Maa, Yangliu Wua, Lu Kanga, Quanshun Ana, Jingbang Zhanga, Kailing Dengb, Jia-Qi Li[*](#_bookmark7),a and Canping Pan[*](#_bookmark7), a

^a^ Innovation Center of Pesticide Research, Department of Applied Chemistry, College of Science, China Agricultural University, Beijing 100193, China

^b^ Institute of Food Science and Technology, Chinese Academy of Agricultural Sciences, Beijing 100193, China

*Corresponding authors

Canping Pan: [canpingp@cau.edu.cn](mailto:canpingp@cau.edu.cn); jiaqili@cau.edu.cn

Total number of pages: 8

Total number of figures: 2

Total number of tables: 4

**SUPPORTING TABLES AND FIGURES**

**Table S1.** UPLC-MS/MS parameters of plant hormones

**Figure S1.** GO class of different treatments in the root

**Table S2.** UPLC-MS/MS parameters of lignin-related metabolites

**Figure S2.** GO class of different treatments in the leaves

**Table S3.** Primer sequences for qPCR

**Table S4.** Statistics of the number of differentially expressed genes

**Table S1.** UPLC-MS/MS parameters of plant hormones

| **Name** | **Precursor/quantitative ion (m/z)** | **Precursor/qualitative ion (m/z)** | **Retention time (min)** | **Collision energy (CE) (V)** | **Polarity** | **Delta EMV(-)** |
| --- | --- | --- | --- | --- | --- | --- |
| **JA** | 209.2/59.1 | 209.2/209.2 | 1.81 | 5/5 | Negative | 500 |
| **SA** | 137.1/93.1 | 137.1/137.1 | 1.64 | 5/15 | Negative | 500 |
| **ABA** | 263.3/153.1 | 263.3/219.2 | 1.75 | 10/10 | Negative | 500 |
| **BL** | 481.4/445.1 | 481.4/315.1 | 4.37 | 4/10 | Positive | 500 |


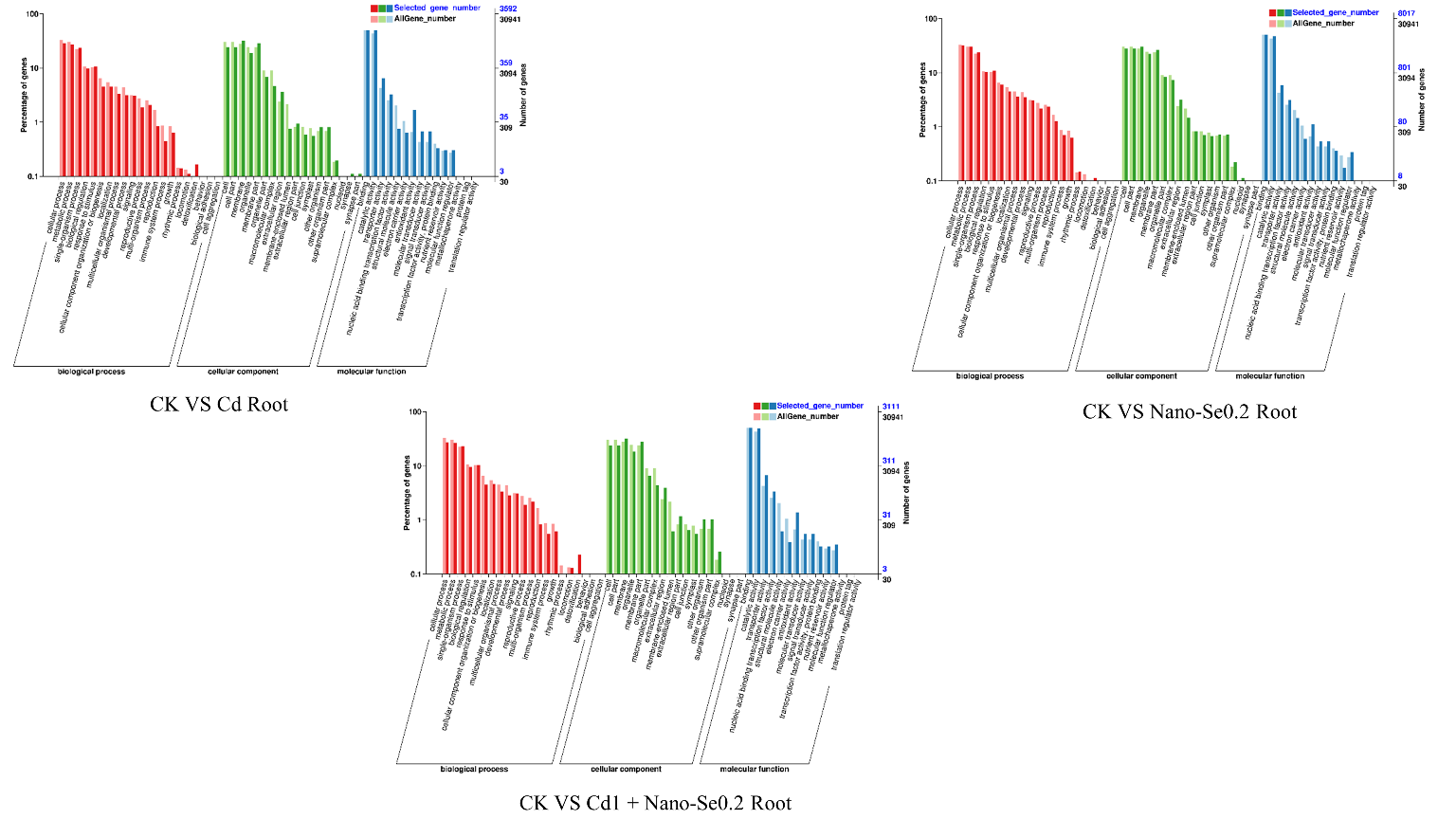


**Figure S1.** GO class of different treatments in the root

**Table S2.** UPLC-MS/MS parameters of lignin-related metabolites

| **Name** | **Precursor/quantitative ion (m/z)** | **Precursor/qualitative ion (m/z)** | **Retention time (min)** | **Fragmentor(F)(V)** | **Collision energy (CE) (V)** | **Polarity** |
| --- | --- | --- | --- | --- | --- | --- |
| *p*-coumaric acid | 163.1/119 | 163.1/163.1 | 0.39 | 90 | 20/20 | Negative |
| coniferaldehyde | 179.1/91.1 | 179.1/119 | 0.66 | 90 | 30/20 | Positive |
| *p*-coumaraldehyde | 149.1/131 | 149.1/103 | 1.27 | 90 | 10/20 | Positive |
| phenylalanine | 180.1/120 | 180.1/163.1 | 1.03 | 90 | 10/10 | Positive |
| cinnamic acid | 147.1/103 | 147.1/62.2 | 0.61 | 60 | 10/10 | Negative |
| *p*-Coumaryl alcohol | 151/110 | 151/128.1 | 2.39 | 60 | 4/4 | Positive |
| caffeyl alcohol | 167.2/84.9 | 167.2/126 | 2.51 | 60 | 4/10 | Positive |
| sinapyl alcohol | 153.1/111.9 | 153.1/67.7 | 1.16 | 60 | 4/10 | Positive |


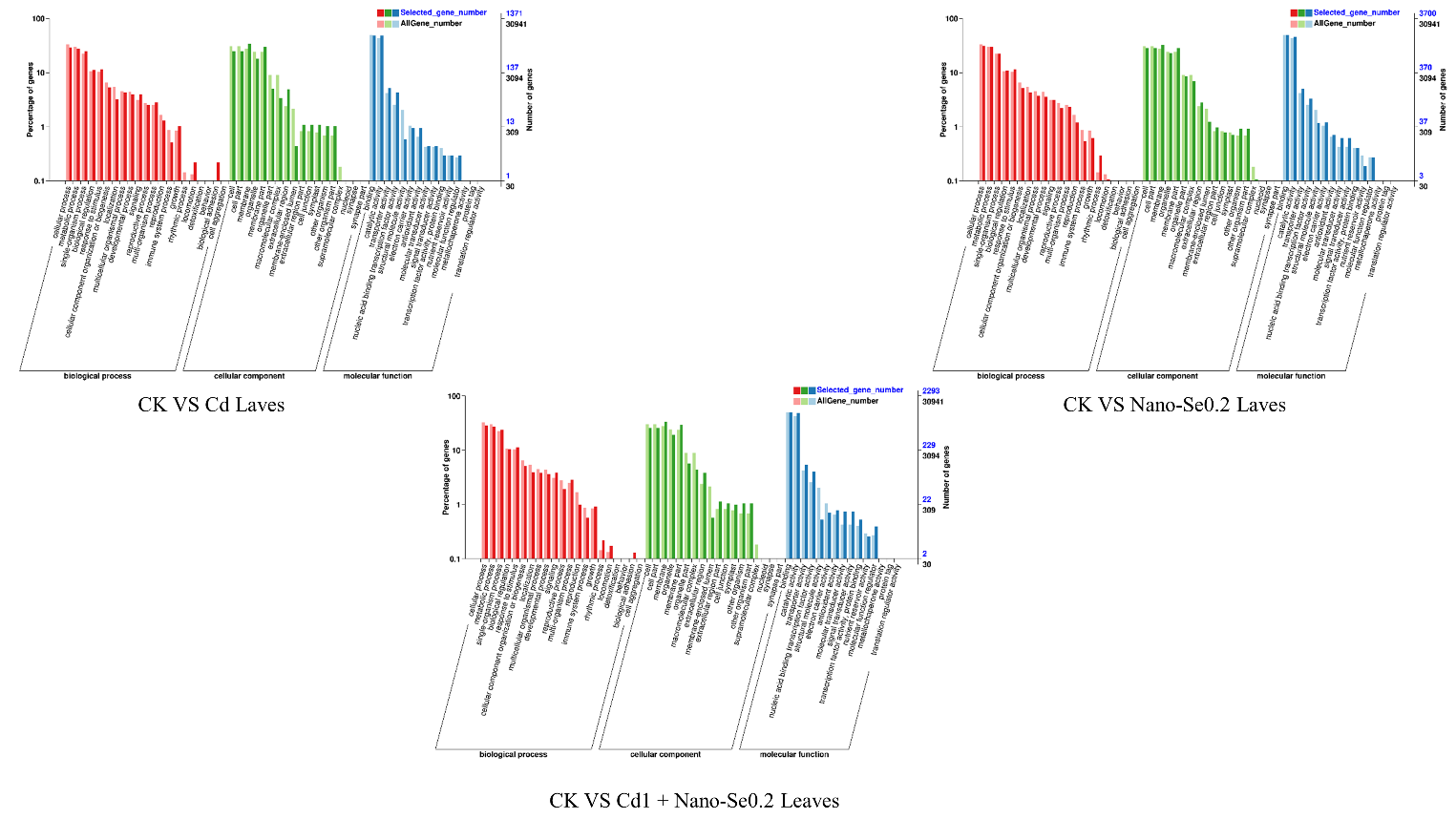


**Figure S2.** GO class of different treatments in the leaves

**Table S3.** Primer sequences for qPCR

| Gene | Abbreviation | Sequence |
| --- | --- | --- |
| Phe ammonia lyase | *PAL* | Forward: ATTCGCGCTGCAACTAAGAT Reverse:CACCGTGTAAGGCCTTGTTT |
| Coumaric acid 3-hydroxylase | *C3H* | Forward: GCCATCTTCTGCACCATTTT  Reverse: GGCCTGTAATGGAGTCCTCA |
| Hydroxycinnamoyl transferase | *HCT* | Forward: ATGCAGGGATGAAGATGGAC  Reverse: TAATCAACGGCCGGAATAAG |
| Caffeic acid O-methyltransferase | *COMT* | Forward: CCTGCGAATGGAAAAGTGAT  Reverse: TCTTTGCCTCCTGGGTTATG |
| 4-coumarate: CoA ligase | *4CL* | Forward: TCCATCGGCATCTTCGATAGT  Reverse: TCAACTTCAGGACACCACCG |
| Cinnamyl alcohol dehydrogenase | *CAD* | Forward: CTGGTTGGCTTCCCAAGTGA  Reverse: TACCTGCAAGAGGAGAGCCA |
| Brassinosteroid signaling positive regulator | *BZR1* | Forward: CCGAGCTTGCTAGCTGATGA  Reverse: CCCAAACTAAACTTAAAGGCCTCA |
| Lipoxygenase 3 | *LOX3* | Forward: TGGTGATCCTGCGAATGGTT  Reverse: CGTCCCAATCAAACGTGACA |
| Isochorismate synthase 2 | *ICS2* | Forward: CGCCTTACAGCTCTGGCACTA  Reverse: CGAGGGAGGAGCAGATGGT |
| 9-cis-epoxycarotenoid dioxygenase | *NCED1* | Forward: GAAAAGGAATGGAAATCGGA  Reverse: CGGGGACGTATATTCTAAAC |

**Table S4**. Statistics of the number of differentially expressed genes

| **#Parameters** | **Group** | **DEGs_total** | **DEGs_up** | **DEGs_down** |
| --- | --- | --- | --- | --- |
| Software：DESeq2_edgeR | L01&L02&L03&L04_vs_LC01&LC02&LC03&LC04 | 1888 | 862 | 1026 |
|  | L01&L02&L03&L04_vs_LCS01&LCS02&LCS03&LCS04 | 3204 | 1475 | 1729 |
|  | L01&L02&L03&L04_vs_LS01&LS02&LS03&LS04 | 5187 | 2794 | 2393 |
|  | R01&R02&R03&R04_vs_RC01&RC02&RC03&RC04 | 4975 | 2486 | 2489 |
|  | R01&R02&R03&R04_vs_RCS01&RCS02&RCS03&RCS04 | 4296 | 2061 | 2235 |
|  | R01&R02&R03&R04_vs_RS01&RS02&RS03&RS04 | 11080 | 5688 | 5392 |
